# Glaucoma surgery and induced astigmatism: a systematic review

**DOI:** 10.1186/s40662-017-0090-x

**Published:** 2017-11-17

**Authors:** Helen H. L. Chan, Yu Xiang G. Kong

**Affiliations:** Royal Victorian Eye and Ear Hospital, 32 Gisborne St, East Melbourne, VIC 3002 Australia

**Keywords:** Filtration surgery, Trabeculectomy, Astigmatism, Refractive outcome

## Abstract

**Background:**

The refractive outcomes of glaucoma surgeries, particularly their effect on astigmatism, are incompletely understood.

**Main body:**

Trabeculectomy is associated with a considerable amount of with-the-rule astigmatic change in the immediate postoperative period. This is followed by a gradual against-the-rule shift. These changes are altered with the use of mitomycin C (MMC). Non-penetrating surgery such as deep sclerectomy is also associated with a similar or smaller degree of induced astigmatism. Minimally invasive glaucoma surgery appears to be astigmatically neutral. There is no clear evidence regarding refractive outcomes of glaucoma drainage device surgery.

**Conclusions:**

Induced astigmatism may account for a reduction in unaided visual acuity in the early postoperative period following a successful trabeculectomy. These changes appear to stabilise at 3 months, and it would be prudent to defer the prescription of new glasses until this time. If sequential cataract surgery is to be performed, toric intraocular lenses can be a useful option for astigmatic correction.

## Background

Glaucoma is the leading cause of irreversible blindness globally [1]. Glaucoma surgery is necessary when maximally tolerated medical therapy is not sufficient to control disease progression [2]. Trabeculectomy is the most commonly performed glaucoma operation [3]. Other surgical techniques include glaucoma drainage devices, non-penetrating surgery such as deep sclerectomy, and newer minimally invasive glaucoma surgery. Despite the popularity of such procedures, the impact of glaucoma surgical techniques on refraction, particularly astigmatism, are incompletely understood. It is common for patients undergoing glaucoma surgical procedures to have a brief period of reduced visual acuity in the early postoperative period, and for some patients, this can persist long-term. While the change in vision can be due to the reduction in intraocular pressure (IOP), several studies have examined the direct effect of glaucoma surgery on astigmatism, suggesting that changes to corneal topography and refractive status can play a role. The purpose of our review is to summarise the existing literature on the effect of glaucoma surgery on astigmatism and its significance for vision rehabilitation.

## Main text

### Type and amount of induced astigmatism

#### Trabeculectomy

Hugkulstone was the first to examine corneal astigmatism following trabeculectomy [4]. Keratometry was performed pre-operatively and at 1, 3, 14 and 28 days post-operatively in 10 patients who underwent trabeculectomy. He reported a statistically significant reduction in vertical corneal radius from a mean of 7.71 mm preoperatively to 7.36 mm at day 1. This steepening of the vertical meridian, that is, with-the-rule astigmatism (WTR), was observed at all post-operative time points up to the last follow-up at 28 days. A corresponding increase in horizontal corneal radius was transiently observed but disappeared by day 14. There was no significant alteration in the angle of the steep meridian. Of note, Hugkulstone found no correlation between the keratometric measurements and IOP. He also did not observe any correlation between the changes in corneal radii and final visual acuity and concluded that the induced astigmatism did not appear visually significant.

These findings were replicated by Cunliffe et al., who measured refraction and keratometry in 16 patients undergoing trabeculectomy [5]. Vertical corneal radius was reduced from a preoperative mean of 7.69 mm to 7.56 mm at week 1. This WTR change in corneal astigmatism persisted at week 3 and 8 of follow-up but had returned to preoperative values at final follow-up at 10 months. Horizontal corneal radius was increased at weeks 1 and 3 however, had normalised from week 8 onwards. They reported no difference in the axis of the vertical corneal curvature. In addition, Cunliffe et al. demonstrated a myopic shift related to altered anterior chamber depth (ACD), with a 1 mm change in ACD giving approximately 2 diopters (D) spherical change. They proposed that the induced refractive error, i.e., myopia and astigmatism, accounted for the reduced unaided visual acuity commonly observed in the immediate postoperative period.

Later, Rosen et al. extended these findings when comparing corneal topography and keratometry in 8 eyes from 6 patients undergoing trabeculectomy [6]. They confirmed a WTR shift of 1.5 to 2.5 D of cylinder up to 12 weeks post operatively and further reported that the amount of induced steepening was underestimated by keratometry compared to topography.

In a larger study measuring corneal topography in 29 patients undergoing trabeculectomy, Claridge and colleagues observed more complex changes [7]. Although they confirmed an overall trend towards vertical steepening and WTR astigmatism, they identified 3 subgroups characterised by 1) superior corneal steepening, 2) superior corneal flattening, and 3) irregular changes. They also demonstrated that in some patients, the topographic changes induced by surgery are present for up to 12 months.

### The role of mitomycin C

More recent studies have examined corneal astigmatism following augmented trabeculectomy. Use of mitomycin C (MMC) appears to be associated with longer lasting astigmatic change.

Hong et al. compared 18 eyes undergoing trabeculectomy with MMC, to 14 eyes having trabeculectomy without MMC at 1 week, 1, 3 and 12 months postoperatively [8]. Use of MMC was associated with less induced WTR astigmatism in the early postoperative period (1.01 D with MMC compared to 2.63 D without MMC at 1 month). Both groups subsequently underwent a further against-the-rule (ATR) change, however, the group without MMC stabilised at 3 months, while the group with MMC continued to experience ATR shift to time of final follow-up at 12 months (0.22 D with MMC versus 1.24 D without MMC at 3 months; −0.34 D with MMC versus 1.42 D without MMC at 12 months).

Further, Hong and colleagues compared 5 eyes having the MMC-augmented triple procedure (extracapsular cataract extraction and trabeculectomy) to 9 eyes having the triple procedure without MMC. Use of MMC was associated with a smaller amount of induced WTR astigmatism at 1 week postoperatively (1.81 D versus 4.50 D) however, both groups were similar by 1 month (1.41 D versus 1.13 D). The triple procedure/MMC group continued to exhibit ATR shift up to time of last follow-up at 12 months, while the amount of induced astigmatism in the triple procedure/no MMC group stabilised at 3 months (−0.49 D with MMC versus 0.05 D without MMC at 3 months; −1.73 D with MMC versus 0.13 D without MMC at 12 months). Hong et al. postulated that the induction of WTR astigmatism was due to wound healing and that use of MMC may reduce the amount of induced astigmatism by inhibition of fibroblast proliferation and other effects on the wound healing process.

Use of MMC may also affect the duration of observable astigmatic change following glaucoma surgery. Kook et al. examined 18 eyes of 16 patients at 1, 3, 6, and 12 months following trabeculectomy using MMC [9]. A concentration of 0.4 mg/mL was applied for 2 to 5 min. Confirming previous studies, they found a mean WTR change in corneal astigmatism of 1.23 D at 3 months postoperatively, followed by a period of ATR change. Interestingly, changes in corneal astigmatism were still present at 6 and 12 months (0.94 D and 0.65 D respectively), much longer than previously demonstrated without MMC. All eyes were found to conform to this pattern of early WTR followed by persisting ATR change, regardless of whether they had WTR or ATR astigmatism preoperatively.

In contrast, a larger study of 47 eyes demonstrated earlier resolution of astigmatic change with MMC use. Delbeke et al. reported only a small mean WTR change in corneal astigmatism of 0.35 D at 1 month, which had almost disappeared at 3 months (0.18 D) and remained stable at 6 months postoperatively [10]. MMC was used at a concentration of 0.2 mg/mL for 2 min, compared to 0.4 mg/mL for 2 to 5 min, and the authors suggested that this lower dose and exposure time accounted for the differences in residual MMC action on wound healing.

### Ex-press miniature glaucoma implant

Ex-PRESS Miniature Glaucoma Implant surgery (Alcon Inc.) is a commonly performed minimally invasive glaucoma surgery. While its implantation also requires formation of a scleral flap and conjunctival suturing, the surgery appears to be astigmatically neutral in the long term. Hammel and colleagues examined 19 eyes undergoing Ex-PRESS implant surgery with Pentacam at regular intervals up to 3 months postoperatively [11]. They reported a transient increase in both anterior and posterior corneal astigmatism on day 1 (from 2.6 to 4.7 D anterior corneal astigmatism; 0.4 to 0.9 D posterior corneal astigmatism) that resolved by the time of follow-up at 3 months. Of note, they demonstrated that the anterior and posterior corneal astigmatism were correlated with IOP and ACD, suggesting that the short-lived corneal changes were due to IOP fluctuations.

### Glaucoma drainage device

To our knowledge, there are presently no studies examining refractive outcomes following glaucoma drainage device (GDD) surgery.

Francis et al. studied axial length following Baerveldt tube implantation but did not specifically examine refractive outcomes [12]. They found a variable reduction in axial length dependent on the amount of IOP reduction and final postoperative IOP. They emphasised that this alteration in axial length must be taken into account when considering intended refractive outcome, particularly in conjunction with cataract surgery as changes in axial length postoperatively can lead to refractive surprise.

More recently, Tzu et al. examined refractive outcomes in combined phacoemulsification and glaucoma surgery (22 eyes combined trabeculectomy, and 21 eyes combined GDD) [13]. 77% of the combined trabeculectomy eyes and 71% of the combined GDD eyes achieved their defined target refractive outcome (between −1.00 D and +0.50 D) at 6 months postoperatively, compared to 85% of a control group who had phacoemulsification alone. Unfortunately, keratometry data was limited to 22 eyes. They found a mean induced astigmatism of 1.31 D in the combined surgery group however, this did not differentiate between the combined trabeculectomy and combined GDD populations.

### Non-penetrating surgery

Egrilmez and colleagues examined 10 eyes undergoing deep sclerectomy with a non-absorbable implant (T-Flux®) [14]. Compared to a trabeculectomy control group, the deep sclerectomy group experienced less induced WTR astigmatism in the early postoperative period and less ATR shift thereafter (0.62 D versus 1.06 D at 3 months; 0.62 D versus 1.24 D at 6 months). They postulated that the thin layer of trabecular meshwork tissue that is left intact in non-penetrating surgery may help reduce the amount of induced corneal steepening.

However, a larger study suggested that both deep sclerectomy and trabeculectomy induced considerable postoperative astigmatism. El-Saied et al. compared 60 eyes undergoing deep sclerectomy with MMC to 60 eyes undergoing trabeculectomy with MMC [15]. They reported that both groups exhibited a statistically significant and similar WTR change in astigmatism of 0.67 D in the deep sclerectomy group and 0.82 D in the trabeculectomy group at 6 months postoperatively.

In addition, Corcostegui et al. studied refractive outcomes following combined cataract surgery and deep sclerectomy with an absorbable implant (SKGel®) in 38 eyes and reported that the mean change in astigmatism was less than 0.50 D and not statistically significant [16].

### Minimally invasive glaucoma surgery

Currently, there is insufficient information on the astigmatic effects of other minimally invasive glaucoma surgeries such as iStent (Glaukos), Cypass stent (Alcon) and Xen gel-stent (Allergan). Given iStent is trans-trabecular and Cypass stent drains into the supraciliary space, both with no requirements for conjunctival suturing and antimetabolite application, it is expected that it will not lead to astigmatic or refractive changes per se*,* apart from the surgically induced corneal flattening from a temporally placed keratome wound, which often serves as the main wound for a combined phacoemulsification procedure. On the other hand, Xen gel-stents are often inserted with MMC application. While there is no requirement for conjunctival suturing or scleral flap formation, filtration bleb formation is an intrinsic part of the surgery and as such the long-term effects of the operation on corneal topographic changes are unclear.

## Mechanisms of astigmatism induction

The reasons for the WTR astigmatism observed after trabeculectomy are incompletely understood. In fact, Hugkulstone had originally anticipated that surgically-induced wound gape would lead to an increase in vertical corneal radius and therefore ATR change. However, to explain the observed WTR changes, he proposed that the posterior placement of the incision restricted the amount of gape and that the overlying scleral flap and sutures provided support limiting any flattening of the vertical meridian [4].

Cunliffe et al. postulated that the removal of tissue (2 × 4 mm block) from under the scleral flap allowed the corneal edge of the trabeculectomy opening to sink slightly, causing a steepening in the vertical corneal meridian [5].

The role of sutures and suture lysis in astigmatism induction has been discussed in the literature. Dietze suggested that over-tight scleral flap sutures at 12 o’clock could cause local tissue compression and therefore steepening in the vertical meridian [17]. However, Delbeke et al. found no difference in astigmatism between groups that had undergone laser suture lysis of scleral flap sutures (19 eyes in the first week, 17 eyes in the second week), and those that did not undergo suture lysis (of a total of 47 eyes) [10]. Rosen et al. also demonstrated that suture lysis of the scleral flap sutures had no effect on corneal astigmatism [6]. Instead, they suggested that excessive cautery may have caused scleral contraction and therefore steepening in the meridian of surgery.

Claridge et al. agreed that this superior steepening could be attributed to a combination of excessive cautery or over-tight sutures, and suggested that this could be further influenced by a large drainage bleb or induced ptosis [7]. Indeed, the pressure of the eyelid and bleb on the cornea may cause changes to the corneal curvature that are dependent on IOP, especially in the early postoperative period [10].

The influence of IOP and hypotony on induced postoperative astigmatism is of particular interest. Kook et al. found a statistically significant correlation between lower postoperative IOP and reduced postoperative axial length (R^2^ = 0.40, *P* = 0.000) in their study of 18 eyes following trabeculectomy with MMC [9]. However, they did not specifically report on the relationship between postoperative IOP and astigmatism.

Interestingly, Delbeke et al. demonstrated a significant negative correlation between postoperative IOP and astigmatism in their study of 47 eyes undergoing trabeculectomy with MMC [10]. They reported a correlation between lower IOP and greater amounts of induced WTR astigmatism at 1 month postoperatively (R^2^ = −0.49, *P* = 0.001). An IOP of 3 was associated with a WTR change of approximately 1 D, whereas an IOP of 11 was astigmatically neutral (Delbeke et al., Fig. 2). This correlation was not present at 6 months postoperatively (R^2^ = −0.07, *P* = 0.656). Delbeke and colleagues hypothesised that the eye is likely more susceptible to deformation and therefore greater astigmatic changes during times when the IOP is lowest.

## Intraoperative steps to avoid astigmatism induction

Several modifications to intraoperative technique have been proposed to reduce the amount of astigmatism induced during trabeculectomy. These steps aim to mitigate the causes of astigmatism postulated above.

A smaller sclerostomy has been advocated to reduce wound gape and “sinking” due to tissue removal [4, 5]. This can be achieved by use of a standardised punch such as a Kelly punch, using a “one punch only” technique, or even implantation of an Ex-PRESS shunt. Care may be taken to ensure a narrow, short scleral flap and to keep the limbal ring intact when making the radial cuts [18, 19].

Scleral flap sutures that are over-tight or of unequal tension should be avoided [17, 18, 20].

Excessive cautery may be limited by reducing the energy, or use of a point-tip or wet field cautery, particularly at the limbus [6, 10, 18, 19].

As with all trabeculectomy procedures, overhanging and intracorneal dissection of the bleb should be avoided as this can cause significant astigmatism and reduced visual acuity long-term. This may be helped by the use of a fornix-based rather than limbus-based conjunctival flap, and broader, more posterior application of MMC [7, 10, 21].

The creation of corneal grooves in which to embed conjunctival closure sutures did not appear to influence postoperative astigmatism [22].

An aberrometer may even be used to assess any induced corneal changes intraoperatively [18].

## Postoperative management of induced astigmatism

Refraction may explain some of the reduction in unaided visual acuity observed following trabeculectomy. It is useful to know that the induced astigmatism tends to stabilise, if not completely resolve, by 3 months postoperatively. It is therefore prudent to advise patients of an anticipated waiting period at least 3 months prior to a stable refraction [10, 22]. Additionally, after this waiting period, the amount of residual astigmatism may not be significant enough to affect visual acuity.

If astigmatic correction is required, the prescription of new glasses is a useful option. Contact lens wear is best avoided following trabeculectomy due to the possible increased risk of infection [23].

Sequential cataract surgery, following a suitable interval after trabeculectomy and careful assessment of bleb function, allows the option of astigmatic correction with the implantation of a toric intraocular lens (IOL) [24]. However, toric IOLs may be less suitable for patients with pseudoexfoliative glaucoma due to the risk of capsular bag decentration [25].

Incisional corneal surgery, such as limbal relaxing incisions delivered by femtolaser laser, is another option. However, this is associated with a transient elevation in IOP that is higher in glaucomatous compared to non-glaucomatous eyes [26]. It may be prudent to avoid femtosecond laser treatment in glaucomatous eyes, particularly if a contact applanation patient interface is used [27].

Similarly, if laser corneal astigmatism management is chosen, PRK (photorefractive keratectomy) may be preferred over LASIK (laser-assisted in-situ keratomileusis) to avoid the IOP spike during LASIK flap creation [24]. Following the procedure, one must also consider the impact of reduced corneal thickness on subsequent IOP measurements.

These strategies may be more difficult to employ during combined surgery. IOL power calculations must take into account the variable reduction in axial length observed following combined cataract extraction and trabeculectomy or GDD (dependent on postoperative IOP) [9, 12]. It may be helpful to set a slightly myopic target refraction [24]. Some authors suggest avoiding the use of toric IOLs during combined surgery altogether due to the difficulty in predicting the variable amount of subsequent astigmatism [28, 29]. Doctors need to be diligent in preoperative counselling regarding refractive outcomes after combined surgery, particularly in the initial postoperative period. Complications, such as hypotony, may cause even greater alterations in axial length and induced astigmatism [29].

## Conclusions

Trabeculectomy can cause a significant amount of induced astigmatism. This may partially account for the reduction in unaided visual acuity commonly observed.

WTR change occurs in the early postoperative period, followed by gradual ATR shift. These effects can be modulated with the use of MMC and its action on the wound healing process and may depend on the concentration and duration of application.

These changes appear to stabilise at 3 months, and it would be prudent defer permanent astigmatic correction until that time.

Deep sclerectomy can be associated with a similar or smaller degree of induced astigmatism. MIGS appears to be astigmatically neutral. There is no clear evidence regarding the astigmatic outcomes following glaucoma drainage device surgery.
